# Exploring How Youth Use TikTok for Mental Health Information in British Columbia: Semistructured Interview Study With Youth

**DOI:** 10.2196/53233

**Published:** 2024-07-05

**Authors:** Roxanne Turuba, Willow Cormier, Rae Zimmerman, Nikki Ow, Marco Zenone, Yuri Quintana, Emily Jenkins, Shelly Ben-David, Alicia Raimundo, Alessandro R Marcon, Steve Mathias, Jo Henderson, Skye Barbic

**Affiliations:** 1 Department of Occupational Science and Occupational Therapy University of British Columbia Vancouver, BC Canada; 2 Foundry Providence Health Care Vancouver, BC Canada; 3 Health Law Institute University of Alberta Edmonton, AB Canada; 4 Division of Clinical Informatics Beth Israel Deaconess Medical Centre Boston, MA United States; 5 Harvard Medical School Boston, MA United States; 6 Homewood Research Institute Guelph, ON Canada; 7 School of Nursing University of British Columbia Vancouver, BC Canada; 8 School of Social Work University of British Columbia Vancouver, BC Canada; 9 Centre for Advancing Health Outcomes Vancouver, BC Canada; 10 Centre for Addiction and Mental Health Toronto, ON Canada; 11 Department of Psychiatry University of Toronto Toronto, ON Canada; 12 Youth Wellness Hubs Ontario Toronto, ON Canada

**Keywords:** youth, adolescents, young adults, mental health, TikTok, social media, qualitative research

## Abstract

**Background:**

TikTok (ByteDance) experienced a surge in popularity during the COVID-19 pandemic as a way for people to interact with others, share experiences and thoughts related to the pandemic, and cope with ongoing mental health challenges. However, few studies have explored how youth use TikTok to learn about mental health.

**Objective:**

This study aims to understand how youth used TikTok during the COVID-19 pandemic to learn about mental health and mental health support.

**Methods:**

Semistructured interviews were conducted with 21 youths (aged 12-24 years) living in British Columbia, Canada, who had accessed TikTok for mental health information during the COVID-19 pandemic. Interviews were audio-recorded, transcribed verbatim, coded, and analyzed using an inductive, data-driven approach.

**Results:**

A total of 3 overarching themes were identified describing youth’s experiences. The first theme centered on how TikTok gave youth easy access to mental health information and support, which was particularly helpful during the COVID-19 pandemic to curb the effects of social isolation and the additional challenges of accessing mental health services. The second theme described how the platform provided youth with connection, as it gave youth a safe space to talk about mental health and allowed them to feel seen by others going through similar experiences. This helped normalize and destigmatize conversations about mental health and brought awareness to various mental health conditions. Finally, the last theme focused on how this information led to action, such as trying different coping strategies, discussing mental health with peers and family, accessing mental health services, and advocating for themselves during medical appointments. Across the 3 themes, youth expressed having to be mindful of bias and misinformation, highlighting the barriers to identifying and reporting misinformation and providing individualized advice on the platform.

**Conclusions:**

Findings suggest that TikTok can be a useful tool to increase mental health awareness, reduce stigma, and encourage youth to learn and address their mental health challenges while providing a source of peer connection and support. Simultaneously, TikTok can adversely impact mental health through repetitive exposure to mentally distressing content and misleading diagnosis and treatment information. Regulations against harmful content are needed to mitigate these risks and make TikTok safer for youth. Efforts should also be made to increase media and health literacy among youth so that they can better assess the information they consume online.

## Introduction

TikTok is a social media platform that is mainly used by youth and young adults younger than 30 years of age to create and share short videos [1]. TikTok was created as a music-based entertainment app that involved lip-syncing and dancing [2] but has since evolved as a way to share information about diverse topics, ranging from news coverage [3,4] and politics [4,5] to public health [6] and social justice issues [3,4,7]. TikTok uses a complex algorithm to recommend content based on viewers’ interactions with previous posts, such as viewing time, likes, shares, and comments, resulting in content catered to each individual’s interests [8]. This content floods users’ “For You” page, which runs through an automated loop that users can scroll through.

TikTok experienced a surge in popularity during the COVID-19 pandemic, providing youth with a platform to interact with each other and share their lived experiences and thoughts related to the pandemic, including social isolation, public health measures, and ways to cope with mental health challenges [9-11]. TikTok has also been used to incorporate humor when sharing experiences in the face of psychological distress, which can facilitate coping and social connection [12]. For example, the “stupid walk challenge,” where TikTok users would refer to “going on a stupid walk for my stupid mental health,” became a popular way to share the common struggle of maintaining mental well-being during social isolation [13]. The hashtag #MentalHealth also became popular, with over 100 billion views on TikTok as of August 2023, with users sharing their mental health journeys and encouraging conversations about mental health [13]. Although this proliferation of social media content has increased mental health awareness [14], there have been growing concerns about the quality of health advice being shared on the platform [15] and the impact of persistent exposure to mentally distressing content [6]. A recent content analysis [6] found that while most TikTok videos using the hashtag #MentalHealth were filled with supportive and validating comments, almost half of these videos reported or expressed symptoms of mental distress, which could have concerning effects on viewers’ mental health.

With youth spending a significant amount of time on the platform—on average, TikTok users spend 95 minutes per day on the app [16]—there is a need to better understand the types of information youth are accessing and how it impacts their mental health. In British Columbia, 26% of youths (ages 12-19 years) reported having a mental health condition in 2018, with anxiety (19%) and depression (15%) being the most common [17]. The COVID-19 pandemic greatly exacerbated youth’s mental health issues [18-20], which was reflected in increased emergency department visits and hospitalizations across Canada for suicidal ideation, self-poisoning, and self-harm [21]. Many youth have reported that their mental health has worsened during the COVID-19 pandemic, mainly due to social isolation, missing out on life experiences, fear of getting sick, and challenges accessing mental health services [18,20].

Although TikTok has become a popular platform for mental health information, very little research has explored how youth use TikTok to learn about mental health and how the COVID-19 pandemic influenced this behavior. As such, this study aimed to address the following research question: How did youth use TikTok during the COVID-19 pandemic to access information and support about mental health? This is one of the first studies to explore the experiences and perceptions of youth accessing mental health information on TikTok through qualitative inquiry.

## Methods

### Study Design and Setting

We conducted a qualitative interview study with youth across British Columbia, Canada. This paper follows the Standards for Reporting Qualitative Research (SRQR) [22]. British Columbia is a province on Canada’s west coast, with approximately 5.4 million people. A total of 88% of the population resides in a metropolitan area, while the remaining 12% live in rural and remote communities across a large mass of land, which limits the availability of mental health services in rural areas.

### Ethical Considerations

This study was reviewed and approved by the University of British Columbia Behavioural Research Ethics Board (REB #H21-02948). Eligible youth were sent a copy of the study information sheet and consent form. All participants met with the research coordinator (author RT) and a research assistant to go over the consent form provided verbal informed consent over Zoom (Zoom Video Communications). The research team member filled out the consent form with each participant over Zoom and sent them a final copy for their records. To protect the participants’ identity, each youth was assigned a participant ID number which was used to deidentify the collected data. The consent forms containing participants’ names were stored separately on an encrypted USB key stored in a locked cabinet at the principal investigator’s office at the University of British Columbia (UBC). All participants received a CAD $30 (US $23.27) honorarium to acknowledge their contributions.

### Sampling and Recruitment

We recruited youth between the ages of 12-24 years who lived in British Columbia, Canada, spoke English, and had accessed TikTok for mental health information during the COVID-19 pandemic. This age range was chosen to reflect the largest population using TikTok (13-30 years of age) [1] and the age criteria for youth services in British Columbia, which typically ends at 25 years of age. Youth were recruited through social media channels belonging to Foundry, a province-wide network of integrated youth services that provides mental health care, substance use services, physical and sexual health, peer support, and social services to youth aged 12-24 years. Foundry offers on-site and app-based services and has had over 250,000 youths visit since 2018. Foundry centers across the province and other partnering mental health organizations reshared the social media posts for the study. Interested youth emailed the research coordinator (author RT), which was followed by a brief screening call to confirm their eligibility.

### Data Collection

Data were collected between June and August 2022. Semistructured interviews were held over Zoom and lasted between 30 and 60 minutes. Participants were given the option to use a different name on Zoom and keep their cameras off during the interview to further protect their identity. The interviews were audio-recorded and transcribed via Zoom and saved on a secure UBC-licensed Zoom cloud server. The research team revised and anonymized the interview transcripts and saved them to a secure UBC server and folder, while the audio recordings were deleted. A research assistant facilitated the interviews, while another research team member took general notes during the interview. The research assistants (authors WC and RZ) were undergraduate students close in age to most of the study participants, which helped to build rapport with the interviewees. They also had their own experience with using the TikTok platform and accessing mental health content on the app, which helped stimulate dialogue during the interviews. Before beginning the interview, participants were asked to complete a short demographic survey distributed through Qualtrics (Qualtrics Developer Platform). Participants were not asked to share their survey responses verbally and were only identified by their unique participant ID number. Throughout data collection, the research team debriefed frequently and discussed whether new topics arose in order to determine whether additional questions should be included in the interview guide to further our understanding of youth’s overall experiences. Interview questions focused on exploring why youth use TikTok for mental health information, the type of content youth access, and the benefits and barriers to using TikTok for mental health information. Participants were also asked how the COVID-19 pandemic impacted their use of TikTok, their accessed content, and their ability to access mental health services.

### Data Analysis

All interviews were audio-recorded, transcribed verbatim, and uploaded to NVivo (version 12; Lumivero) to facilitate analysis. The research coordinator led the analysis using Braun and Clarke’s reflexive thematic analysis method [23,24]. This process began by reading the transcripts and interview notes multiple times while taking additional memos and reflections to support analysis. A data-driven approach was used to generate verbatim codes, which were categorized thematically using a thematic map to visualize and refine. Peer debriefing meetings were held between the research coordinator and 2 youth research assistants (authors WC and RZ) who cofacilitated the interviews to discuss the relationships between the codes and identify potential themes and subthemes. This involved reflecting on our biases stemming from our experience accessing mental health content on social media. The research assistants also supported the selection of quotes that best represented the overarching themes and subthemes.

## Results

### Overview

A total of 21 youths participated in this study. Participants’ median age was 18 (IQR 16-21) years and they primarily identified as women (12/21, 57.1%), bisexual or pansexual (9/21, 42.9%), and White (12/21, 57.1%). Most youth had accessed mental health services before (16/21, 76.2%), including counseling (15/21, 71.4%), prescription medicine (10/21, 47.6%), psychiatry (6/21, 23.8%), peer support (4/21, 19.0%), and case management (3/21, 14.3%; see Table 1).

**Table 1:** Participant characteristics.

Characteristics			Values
**Sociodemographics**			
	**Age (years)**		
		Median (IQR)	18 (16-21)
		Range	13-24
	**Gender identity, n (%)**		
		Woman	12 (57.1)
		Man	5 (23.8)
		Nonbinary	3 (14.3)
		Transgender man	1 (4.8)
	**Sexual orientation^a^, n (%)**		
		Bisexual or pansexual	9 (42.9)
		Heterosexual	6 (28.6)
		Gay or lesbian	1 (4.8)
		Queer	1 (4.8)
		Homoromantic asexual	1 (4.8)
	**Ethnicity^b^, n (%)**		
		White	12 (57.1)
		First Nation, Métis, or Inuit	4 (19.0)
		South Asian (eg, East Indian, Pakistani, and Sri Lankan)	3 (14.3)
		Chinese	2 (6.7)
		Black or African	2 (9.5)
		Latin American	2 (9.5)
		Filipino	1 (4.8)
		Middle Eastern or North African	1 (4.8)
		West Asian (eg, Vietnamese, Cambodian, Laotian, and Thai)	1 (4.8)
	**Living location, n (%)**		
		Fraser Health	7 (33.3)
		Vancouver Coastal Health	7 (33.3)
		Vancouver Island Health	4 (19.0)
		Interior Health	3 (14.3)
	**School or employed, n (%)**		
		Both	11 (52.4)
		School	7 (33.3)
		Employed	2 (9.5)
		Neither	1 (4.8)
	**Highest level of education^a^, n (%)**		
		Some high school	8 (38.1)
		High school diploma	5 (23.8)
		Some college or technical school education	1 (4.8)
		Some university education	2 (9.5)
		Bachelor’s degree	4 (19.0)
	**Current living situation, n (%)**		
		I live with my parents or guardians	16 (76.2)
		I live in an apartment or house independently or with roommates	4 (19.0)
		I live with my partner	1 (4.8)
**Services accessed for mental health (past 12 months)^b^, n (%)**			
	None (I have never gotten treatment for mental health)		5 (23.8)
	Counseling		15 (71.4)
	Prescription medicine		10 (47.6)
	Psychiatry		6 (28.6)
	Peer support		4 (19.0)
	Case management		3 (14.3)
**Type of health service environment accessed for mental health (past 12 months)^b^, n (%)**			
	Family doctor’s office		10 (47.6)
	School counseling services		10 (47.6)
	Foundry center		7 (33.3)
	Private office or clinic		5 (23.8)
	Community health center		4 (19.0)
	Emergency room or department		1 (4.8)

^a^Prefer not to answer (n=1).

^b^Participants could select more than 1 response. Therefore, the number of responses may be greater than the total number of participants who completed the survey.

**Figure 1:**
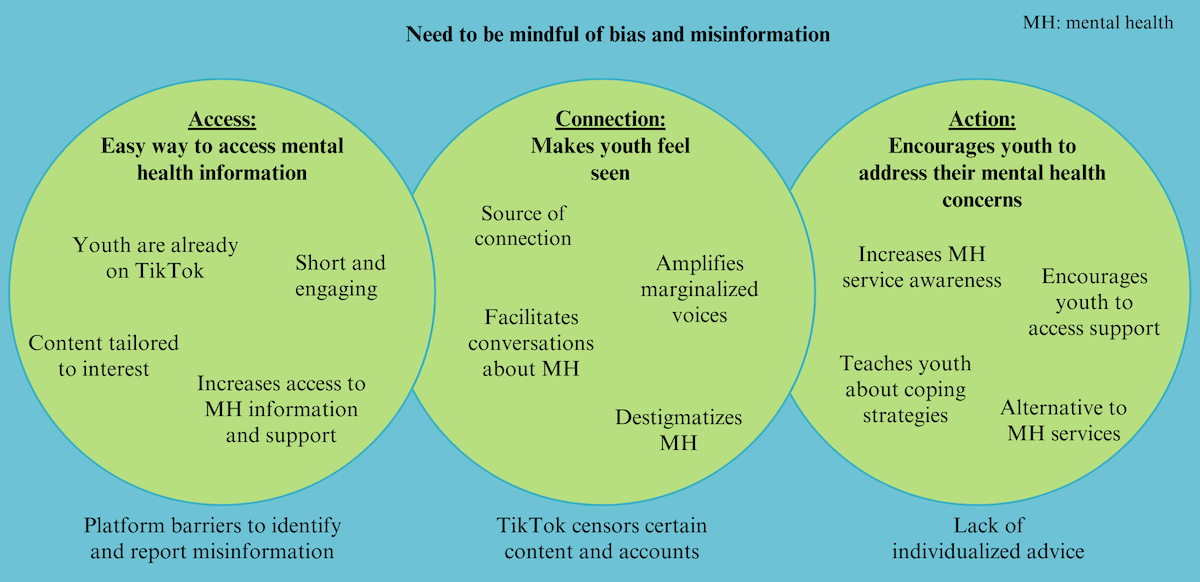
Overarching themes and subthemes describing youth’s experiences using TikTok for mental health information. Across all 3 themes, youth described the need to be mindful of bias and misinformation when consuming mental health information on TikTok. MH: mental health.

A total of 3 overarching and interconnected themes were identified to describe youth’s experiences using TikTok during the COVID-19 pandemic for mental health information and support (see Figure 1). These themes described how TikTok led to increased access to mental health information (theme 1), which in turn increased their connection with others going through similar experiences, making youth feel seen (theme 2). This content led to action, encouraging youth to address their mental health concerns (theme 3). Across these 3 themes, youth highlighted the need to be mindful of bias and misinformation when consuming mental health content on the platform in different ways, which are represented as subthemes under each overarching theme in Figure 1. The following sections summarize each main theme in greater detail, supported by participant quotes.

### Access: TikTok is an Easy Way to Access Mental Health Information

Most youth had joined TikTok for entertainment; however, they began seeing an increase in mental health information on their “For You” page during the COVID-19 pandemic. Most of the youth described how the pandemic exacerbated their mental health challenges, leading them to seek mental health information online. As 1 youth shared:

I guess I started kind of looking at [mental health information] and TikTok ended up having a lot of it during the pandemic because I think a lot of people started searching what they might have when the pandemic kind of exacerbated their issues.

P01

Consequently, youth described how the platform was an important source of mental health information and support during the pandemic. As 1 participant expressed, it was “the perfect way to connect with young people and spread information” (P11), while another participant noted that it made “something that’s quite inaccessible and expensive (i.e., mental health services), free” (P03). Youth were drawn to the content’s short video format and the various ways of presenting information (eg, sketches and using humor) on the platform, which made content engaging, digestible, and relatable. Although many of the youth appreciated informational content on TikTok, they described that they mainly used the platform to disengage from reality and distract themselves from external stressors (eg, social isolation, boredom, and mental health issues). Thus, they were more likely to engage with humorous and trendy content. As 1 youth outlined:

I think I would be more prone to follow somebody if they had a mix of like funny content, where it's like almost following the, you know how there's like waves of trends through TikTok. Like different audios are more popular at certain times. I’d be more inclined to follow somebody if they had a mix of that plus, like, informational stuff and stuff like that, just because when I go on TikTok I’m not necessarily looking to learn as much as I can, I’m just kind of looking to turn my brain off for a little bit.

P19

The use of such engaging strategies was often lacking among the content created by health professionals on TikTok, who were sometimes seen as “kind of boring, like just people saying facts and stuff” (P11). As such, youth tended to gravitate toward content creators with similar experiences and used engaging ways to connect with their audience. Youth also emphasized the benefits of TikTok’s algorithm, as it would tailor mental health information based on what they engaged with and were interested in; however, this also meant that youth who did not engage with mental health content would likely not have access to it. As 1 participant described:

So even if there’s someone who needs mental health support, if it’s not an interest of theirs or it’s not something that they’re engaging with, it may not show up for them.

P09

Further, youth expressed barriers to finding information specific to their location, given the amount of information shared on TikTok and the algorithm’s inability to filter based on region. As 1 youth described:

when TikTok came around, it was like, yeah, there are services. I would get the idea that there are mental health services, but I just didn’t know what they were because these services were from everywhere around the world...I still don’t know to this day, what are the mental health services provided in my area and what can someone do, near me, to help other than my school counsellor.

P20

Some youth described additional challenges accessing mental health services during the COVID-19 pandemic, particularly those who did not enjoy connecting to service providers virtually. As 1 youth explained:

I feel like having that sort of disconnect online is really challenging because it’s hard to trust someone if you only know them through a screen. It made it harder to access personalized services.

P05

Thus, youth appreciated the availability of mental health content on TikTok, as it provided them with alternative mental health support that could be accessed immediately and free of charge. This was also seen as “less intimidating” (P08) than accessing professional services:

It’s like these 30-second clips that—there’s not a whole lot of pressure to engage with. If you want to learn more or if you want to engage, you can, but there’s not necessarily a lot of pressure, like this pressure to go out of your way to follow-up or make an appointment or anything like that.

P09

Access to such content was especially helpful for youth who described having negative mental health care experiences, had been denied mental health support by their parents or caregivers, or simply did not feel comfortable or ready to access mental health support. As 1 participant shared:

It was interesting and helpful hearing people talk about those things...like experiences of autism. That’s not something that I’m really comfortable seeking mental health support for so I am finding it really interesting and helpful, like informally listening to people talk about it and kind of learning more about it and how it might apply to me in that way.

P03

Although youth appreciated having easy access to mental health information via TikTok, youth discussed the importance of being mindful of misinformation because “it’s ultimately all from strangers on the internet” (P06). Youth expressed concerns about content creators boasting professional credentials, particularly counselor influencers whose perceived goal was to gain followers for paid sponsorships. As 1 youth described:

listing their credentials at the beginning of every video that they make, I think it sort of implies to an audience, particularly a not very media critical audience, that this is professional advice, even if they give a little disclaimer like “I’m not trying to give advice, this is just me speaking as a PhD in psychiatry,” like, it kind of offsets their disclaimer when they pump their credentials like that.

P03

Youth highlighted how numerous aspects of the platform exacerbated the spread of misinformation, such as the inability to embed evidence links, short caption sizes, a lack of verified information, and barriers to reporting misinformation, “which made it difficult to tell whether or not something is true” (P15).

### Connection: Mental Health Content on TikTok Makes Youth Feel Seen

TikTok was described as a safe space for people to talk about their mental health experiences without fear of judgment. Many youth appreciated watching TikTok videos about content creators’ mental health experiences (eg, symptoms, coping strategies, and treatments), which helped youth “feel validated” (P21), “know that [they’re] not alone” (P09), and gave “hope that [they] could move forward and get past it” (P04)*.* This was amplified by the wide range of voices (eg, youth, adults, celebrities, and mental health professionals) and mental health topics discussed on the platform. As 1 participant offered:

because of how many different people there are and how wide a range of different mental health issues and topics they cover, and how many different people are affected by mental health differently, it kind of helped me figure out where I can personally work on my mental health and helped me kind of recognize that in myself.

P017

The platform was also described as supporting youth to engage with “various facets of TikTok that are so niche about me and my identity” (P19) and find online communities to which they could relate. This was described as a unique feature found on TikTok compared to other social media platforms. As 1 participant shared:

I liked how it also gave platforms to people who were marginalized and kind of overlooked on other platforms. I saw a lot more of those kinds of people on TikTok than I had on like Instagram, for example, and it kind of made me and a lot of people feel seen.

P01

Youth discussed how the content they accessed on TikTok portrayed mental health concerns as a “common struggle” (P12), which helped normalize and destigmatize conversations related to mental health. Youth described how this was particularly helpful in increasing awareness of certain mental health conditions and experiences that were not often discussed (eg, psychiatric hospitalizations, schizophrenia, and how autism presents in women):

I also like the people who just try and talk about issues that aren’t really talked about much in person. For example, hospitalizations. I don’t see a lot of people talking about their experiences with that because it’s still I think very heavily stigmatized, going to a psychiatric hospital...When people share videos of their stay or what care they received and stuff like that, not only does it destigmatize going to a hospital in general, it also makes people less scared to access that help because a lot of people are scared to go into those sorts of places.

P01

Youth also discussed the “other side” of TikTok, which tended to portray mental health challenges as a personal responsibility rather than something to seek support for. However, this content generally did not reach their For You page as it did not align with the type of information they resonated and interacted with, and thus, was not pushed by the platform’s algorithm. As 1 youth described:

I think it depends on what side of TikTok you’re on, right? So if you’re on mental health TikTok, then it’s very positive, it’s very “you’re not alone”...but if you’re on the quote unquote “wrong side” of TikTok, then you’re going to get a lot of neoliberal “pull yourself up by your bootstraps” attitude.

P12

Youth raised concerns about aspects of the platform that could further contribute to misinformation and stigmatization. For instance, the censoring of specific accounts and mental health content by TikTok moderators’ was described as adding,

to the stigma around mental health...the fact that it can’t be talked about and people can’t access those resources.

P05

For example, 1 youth discussed how content about suicide was often taken down and had to find ways to circumvent the censorship:

when people caption their videos, for example, if they’re trying to talk about suicide...they have to spell it like “seweslid” or something like that. And it’s just like, every time I see that it kind of makes me cringe a little bit because it’s like they’re censoring something that is important to talk about.

P19

A few youth also discussed how TikTok’s algorithm could be “biased against certain demographics and certain topics” (P19) and would often suppress creators who were less “conventionally attractive” (P01) and had disabilities, limiting their access to relevant mental health information.

Youth also expressed concerns about the romanticization of certain mental health conditions and the overgeneralization of symptoms. This could lead to youth pathologizing “normal” human experiences and making inaccurate self-diagnosis. As 1 participant articulated:

There's a lot of room for self-diagnosis on TikTok. Especially if people do like, put a finger down challenge for ADHD [attention-deficit/hyperactivity disorder], and if you get over five, good chance you have ADHD and I’m like, well I have six and I know I don't have ADHD. So there's a lot of room for assumptions and taking in a lot of information that you don't know if it's true or not. Because anybody can post on TikTok, anybody can make a video and post it and you don't know what's true. So that's definitely a negative and you have to be really careful about what you just even subconsciously take in and believe.

P13

Youth underscored how this phenomenon could negatively impact people who are living with a medical diagnosis, as it further stigmatizes and trivializes their lived experiences.

Youth also highlighted aspects of the platform that could negatively impact their mental health. For instance, being regularly exposed to negative experiences and “trauma dumping” (P20) was described as discouraging and took a toll on youth’s mental health. As 1 participant expressed:

it’s kind of an endless cycle...when I just see someone talking about their own experience it kind of takes me into a rabbit hole of just all these things people have gone through, and it’s like now I feel that weight is on my shoulders.

P20

Although many youth described how mental health videos often led to others sharing their experiences and further discussion in the comments section, a few highlighted the damaging impact negative comments could have (ie, trolling). As 1 youth described:

the comments were like really attacking the creators for absolutely no reason, and I just thought it was really vicious to look at every single day.

P05

Youth expressed concerns about the addictive nature of the platform, which led them to spend too much time on TikTok and negatively impact their mental health. As 1 youth described:

it’s really easy to endlessly scroll on the app and I find myself sometimes just not doing the things that are on my to-do list and going on TikTok instead...after I get off of it, I feel really terrible about myself because I wasted so much time.

P10

This led to some youth deleting the app altogether or finding strategies to limit the amount of time they spent on the platform, which became easier as public health restrictions were eased and youth became busier with other things (eg, school, work, socializing with friends, and going outside).

### Action: Mental Health Content on TikTok Encourages Youth to Address Their Mental Health Concerns

Youth described how accessing mental health information on TikTok encouraged them to “be more mindful” (P11) about their mental health challenges and identify ways to mitigate negative symptoms they were experiencing in real time. Most youth described using TikTok as a way to learn coping strategies to reduce stress, anxiety, anger, procrastination, and negative self-talk, including managing panic attacks. Strategies they considered helpful included meditation, breathing exercises, grounding techniques, inner-child work, journaling, and reaching out to friends. Although the youth acknowledged that some strategies might not work for them, they appreciated having the ability to learn and try new things. As 1 participant shared:

I’ve come out with a couple breathing techniques I hadn’t heard of before, or like my partner has ADHD and we’ve tried a couple things and they actually work so...the couple of hack videos that they have on there, like everyone’s different, but if you even come out with one thing that’s new that works for you, I think it’s pretty awesome.

P12

Notably, this required youth to be mindful that “everybody has different experiences, everybody reacts to certain things in different ways” (P05) and that not everything they consumed would apply to their situation since mental health advice provided on the platform cannot be tailored to each individual. This required a certain level of media and health literacy, as 1 youth describes:

I also try not to fall victim to applying everything to myself because I understand that like, you know, a lot of it doesn’t apply to me and that some of the stuff that I can’t—that is said on TikTok is very generalizable and broad definitions of these actual mental illnesses or effects of mental illnesses—so I do try to be aware of that.

P17

As such, youth appreciated content from people with lived experience, as they were “just speaking from experience and what’s worked for them” (P14), and content creators who avoided “single solution-based videos” (P12). As 1 youth described:

There’s a lot of, like, fat overweight women on TikTok and that was really nice because a lot of the time, you know, doctors are like “Oh you’re depressed and you’re fat, so the answer is exercise,” and so seeing like those peer support and those other creators kind of offering alternatives and things like mindful eating and all these other things like not necessarily these crash course single-based solutions was really nice as well.

P12

While youth shared how TikTok could lead to the misdiagnosis of mental health conditions, they also saw the benefit in helping people identify certain behaviors within themselves that they “might not have found otherwise” (P03). This particularly helped those lacking the ability to get properly diagnosed or where sufficient research on a specific mental health condition was lacking (eg, how symptoms of autism present in women), as long as they were mindful that this was not equivalent to a professional diagnosis.

Mental health content shared on TikTok made youth more aware of the types of resources and services available to them and what they should be looking for to address their individual mental health challenges. As 1 participant offered:

if I were to have looked for a counsellor before I went on TikTok and learned more about my own mental health, I definitely would just look for someone who is generally just a good counsellor...but after looking into that, I now know that it’s very important to look for someone specifically, who specializes more in autism and someone who specializes in youth.

P17

This also normalized getting help, made services feel more approachable, and encouraged some youth to access mental health services:

...people on TikTok talking about going to therapy, that persuaded me to go to therapy and then also made me feel a bit more comfortable going to therapy too...I feel like TikTok helped normalize that a lot.

P06

Youth felt that TikTok helped them stay connected with peers, which helped mitigate the mental health impacts of social isolation during the COVID-19 pandemic. Youth described using the platform to share relatable content and initiate conversations about mental health with their peers, parents, and service providers that they may not have had otherwise. For instance, youth described how they would often share TikTok videos with friends, which made it “a lot easier for us to talk about [mental health]” (P20):

It can be a good way to send something to your friend and say like “hey, this is really funny, but at the same time like, yeah this is something that we’re probably both dealing with” and it can be a good tool to facilitate conversations that way I think.

P09

Youth also described accessing content that helped them advocate for themselves during medical appointments and receive appropriate care. As 1 participant stated:

there’s a lot of videos on self-advocacy as well, so I was able to kind of take some tips to my doctor and get a psychiatrist for myself which was really great.

P12

While most youth acknowledged that TikTok did not replace the need for professional services when dealing with “serious” (P20) mental health concerns, they considered it a helpful tool to identify mental health challenges and potential coping strategies. As 1 youth described:

I don’t think TikTok is the best way to deal with a mental health crisis or to deal with...if you’re trying to diagnose yourself. I think it can be almost harmful in those ways, but I do also think that for people who are trying to, you know, figure themselves out more in relation to mental health, that it can be very beneficial in finding coping mechanisms.

P17

As such, TikTok allowed youth to access support in real time by accessing mental health content on the platform, enabling conversations with friends, family, and service providers, and encouraging them to access appropriate services.

## Discussion

### Principal Findings

The COVID-19 pandemic had profound and deleterious mental health impacts, with youth uniquely affected. Our findings describe how the interviewed youth used TikTok as a means of mental health information and support. TikTok was perceived as an easy way to access mental health information, connect with others going through similar experiences, and learn how to address mental health challenges. Such content helped facilitate conversations about mental health with family, friends, and service providers and encouraged youth to access support. This source of mental health support and connection was significant for confronting the negative impacts of the COVID-19 pandemic, such as social isolation, boredom, exacerbated mental health issues, and challenges accessing mental health services. While TikTok was not seen as a replacement for mental health services, it was viewed as a tool to increase awareness, reduce stigma, and encourage people to address their mental health issues. Conversely, many youth expressed concerns over the lack of safeguards to deal with misinformation and the negative mental health effects that can occur when spending too much time on the platform. As such, youth discussed having to be mindful of bias and misinformation when consuming mental health content on TikTok while limiting the time spent on the platform.

### Increasing Youth’s Access to Information and Community

The findings from this study are particularly timely given that youth increasingly rely on online sources (eg, websites, social media, apps, and online communities) for health information, given the ease and anonymity it provides [25,26]. However, prior studies have found that youth have been unsatisfied by the lack of youth-specific information available online [25], which may partly explain why TikTok is replacing Google as a search engine among younger generations as its content is specifically designed for younger audiences [27]. Indeed, a systematic review exploring the barriers and facilitators to youth mental health-seeking behavior [26] found that societal views and attitudes toward mental health were the biggest obstacles preventing youth from accessing mental health services, contributing to their lack of knowledge about mental health and available mental health services. Youth from this study described how TikTok helped them identify and normalize their mental health challenges and encouraged them to access mental health support, demonstrating the platform’s potential for destigmatizing mental health among youth. This also suggests that TikTok may be useful for mental health professionals and organizations to spread accurate mental health information specifically aimed at youth.

Several studies have found that people turn to online communities to reduce anxiety, depression, and feelings of loneliness [28-30]. This was a common coping mechanism for youth during the COVID-19 pandemic when face-to-face interactions were not possible [31,32]. For instance, a qualitative study exploring youth’s perspectives using online sources for health information [25] found that many youth benefited from seeing what others were doing to support their health, which was a source of motivation and inspiration. These findings align with our study results, as youth described feeling less alone when viewing others going through similar experiences. Moreover, TikTok was described as enabling them to connect with communities and try coping strategies that they would not otherwise have accessed.

Online communities can particularly benefit those who experience greater barriers to accessing supports and services (eg, rural and remote communities and marginalized groups), experience social anxiety, or are concerned about being stigmatized [33,34]. For instance, research suggests that people who identify as LGBTQ2S+ (lesbian, gay, bisexual, transgender, queer, two-spirit, plus) experience greater mental health benefits from active social media use compared to cisgender people as it provides them an outlet for self-expression and access to social support they may not have access to offline [35,36]. Participants from our study mainly identified as LGBTQ2S+ (12/21, 57.1%) and also expressed how TikTok helped them find communities they could relate to. While these sentiments were consistent across our sample, these findings support the idea that social media platforms can be particularly beneficial for marginalized individuals. The anonymity provided online can also help youth feel more comfortable sharing personal stories and increase their access to comprehensive information, which may not always be accessible through caregivers or health care providers [25]. For example, another qualitative study exploring youth’s perspectives on using technology for health information found that youth relied on online sources for comprehensive sexual education, as their school education was solely abstinence-based. This resonates with what we heard from youth in this study, who described how TikTok increased their ability to access mental health information unavailable to them when their parents were involved in their care. As such, TikTok presents an opportunity to promote online support services to increase youth’s access to mental health support; however, data privacy remains a concern [37]. Albeit all social media platforms mine personal data for profit, a study exploring data sharing behaviors across social media platforms found that TikTok shared more personal data to third-party entities compared to all other social media platforms and it is unclear what that information is being used for [38]. As such, it is essential that governments develop legislation and regulations to limit TikTok’s ability to collect and share personal information with third-party services, given the amount of sensitive information being collected and shared on the platform. While TikTok implemented new measures in 2023 to protect younger youth on the platform (eg, limiting screen time, direct messages, and comments from strangers) [39], having a caregiver who understands how the platform works and can ensure these measures are in place is vital.

### Mitigating the Negative Effects of TikTok

Although mental health information on TikTok can be helpful, participants from this study raised concerns about the negative mental health effects of spending too much time on the platform, suggesting that youth may benefit from time limits on social media platforms to limit daily usage. Youth also highlighted the lack of safeguards to report and identify misinformation and having to rely on their own judgment to assess the accuracy of the information presented and read through the comments section to corroborate their assumptions. Misinformation is rampant on social media—this became evident during the COVID-19 pandemic, which led to a boom of misinformation about the disease, its transmission, treatment, and prevention [40]. Yeung et al [41] conducted a cross-sectional study that examined the quality of 100 TikTok videos about attention-deficit/hyperactivity disorder and found that 51% of videos (posted mainly by non–health care professionals) were misleading. Similarly, a systematic review exploring online health information-seeking behavior [42] identified a lack of mechanisms to verify misinformation, making it difficult for consumers to determine credibility. Our findings extend this prior research and suggest that these missing safeguards can lead youth to pathologize everyday experiences, contributing to unnecessary stress, anxiety, and self-diagnosis. This can further reinforce existing stereotypes and lead youth to try inappropriate or ineffective treatments without consulting a health care provider which can have detrimental consequences [43].

This study brings into question TikTok’s responsibility as a platform to prevent the spread of misinformation. While incorporating mechanisms to report misinformation on TikTok can help, this responsibility cannot solely lie on users who may not be able to differentiate between credible and misleading information. Currently, social media platforms have opaque processes for reviewing posts for potential harms [44]. As such, regulations are needed on disclosing policies and methods used to identify and remove harmful content [45].

Other ways to help mitigate the spread of misinformation may include more content from health care professionals. While the number of health care professionals sharing mental health content on the platform is increasing, McCashin and Murphy [46] reported that they are less likely to use the full range of features on TikTok and tend to share more serious content. Thus, health care professionals often have lower engagement levels than peers speaking from lived experience [46], which resonates with our study findings. This highlights the need for health care professionals to create more engaging content to maximize the spread of accurate mental health information and educate viewers about media and health literacy and how to identify misinformation. While working collaboratively with youth to create relatable content on TikTok may be a helpful solution for reaching more youth on the platform, this requires significant time, effort, and investment, which may be difficult for many. More research is needed to understand how to create engaging content that gets sufficient rank and visibility with TikTok’s algorithm.

Alternatively, TikTok could provide mental health professionals and organizations with opportunities to highlight their services through sponsored advertisements and location-specific hashtags. This would help address the lack of location-specific information available about mental health services, a noted barrier to acting on what youth learned on the platform. Incorporating media and health literacy education in schools should also be a priority to ensure youth have opportunities to build critical thinking skills to discern the difference between expert advice and anecdotal experiences, given the time youth spend online. Although anecdotal experiences can foster hope and connection and provide practical suggestions, it is important for youth to assess the relevance of these experiences in relation to their unique situation. Another possible approach would be to mandate public service announcements for mental health support on TikTok from legitimate and trusted health care providers and nonprofits so that all viewers have access to reliable mental health information.

While sharing personal stories on TikTok can be therapeutic, these stories can also be traumatic or triggering to those who have experienced similar situations and may not be ready to review this type of content. Little is known about the impact of consuming repetitive traumatic or triggering content online, a common trend on TikTok [6,47], exacerbated by the repetitive nature of TikTok’s algorithm [48]. When users interact with negative content (eg, self-harm and eating disorders), the algorithm will continuously promote this type of content on their For You page. A study by the Centre for Countering Digital Hate [48] analyzed recommended TikTok videos within the first 30 minutes of creating standard youth accounts and found that content on eating disorders came up every 4.1 minutes, while content on self-harm and suicide was shown every 20 minutes. Youth accounts containing the phrase “lose weight” in their usernames were 3 times more likely to see this type of content on their For You page, particularly self-harm and suicide content, which was 12 times more likely to be shown. This can seriously impact youth’s mental health, especially those who are already struggling. For instance, the parents of a 16-year-old from the United States who died by suicide in 2022 found his For You page flooded with distressing content, including the glorification of suicide [49]. These concerns were raised by participants in our study, highlighting the need for further research on the impacts of mentally distressing content online and to identify ways to mitigate these risks, such as developing legislation that regulates harmful content and TikTok’s algorithm.

### Limitations and Future Directions

While the findings from this study provide important implications for future mental health education efforts, there are limitations to consider. Since TikTok’s algorithm tailors content based on users’ interests and interactions, youth from our study all desired access to mental health information. This suggests that we may be missing the perspectives of youth less likely to discuss mental health and access mental health support. This also highlights the potential pitfalls of TikTok’s algorithm, which prevents mental health information from reaching individuals who may benefit from it the most, and the need to incorporate disclaimers linking to evidence-based mental health information to mitigate the spread of misinformation. Further efforts to reach this population are warranted to ensure everyone has access to accurate mental health information and support. Additionally, our findings represent the experiences of youth who mainly identified as White and women. Having a more ethnic and gender-diverse sample could have provided further insights into youth’s use of TikTok for mental health information as minority populations experience different access to mental health care and support compared to White, cisgender individuals [35,36]. While our sample did include many youth who identified as LGBTQ2S+, further research is warranted to identify new opportunities that social media platforms can provide in terms of mental health support for minority groups. While these limitations impact the generalizability of our findings, this is one of the first studies to explore the experiences of youth with TikTok, specifically their experiences accessing mental health information on the platform. The findings provide important direction for future research on the mental health impacts of the platform and its algorithms. They also have important considerations for policy makers shaping legislation regulating social media content to prevent harm and relay accurate mental health information.

### Conclusions

Our findings shed light on how TikTok was an important source of mental health information and support for youth during the COVID-19 pandemic and how it has increased mental health awareness, encouraged youth to address their mental health issues, and reduced the stigma of help-seeking. This study highlights how TikTok can be a valuable tool for youth to access relatable mental health information, peer connection, and support while helping facilitate conversations about mental health between youth and friends, family, and service providers. Conversely, the lack of safeguards to deal with misinformation and harmful content on the platform is concerning and suggests the need for better regulations against harmful content and increased media and health literacy education among youth. Further research on the impact of TikTok’s algorithm and repetitive exposure to mentally distressing content online is needed to mitigate the potential risks associated with the platform.

## Abbreviations

LGBTQ2S+: lesbian, gay, bisexual, transgender, queer, two-spirit, plus
SRQR: Standards for Reporting Qualitative Research
UBC: University of British Columbia
